# Persistent sle activity related to untreated reactivation of pulmonary tuberculosis

**DOI:** 10.1186/1546-0096-12-S1-P331

**Published:** 2014-09-17

**Authors:** Assunta Chi Hang Ho

**Affiliations:** 1 Paediatrics, Prince of Wales Hospital, Chinese University of Hong Kong, Hong Kong, Hong Kong

## Introduction

SLE patients are at risk of tuberculosis (TB) infection due to both secondary immunosuppression and intrinsic defects in innate immunity. On the other hand infection can also perpetuate the development of an autoimmunity [1,2].

## Objectives

Here we report a case of pulmonary tuberculosis reactivation in a SLE patient. The lupus activity was not responsive to heavy immunosuppressants. It was only controlled after commencement of anti TB treatment.

## Methods

WLS was diagnosed SLE at the age of 16 with fever, rash, oral ulcers, cytopenia, lupus nephritis (type II and III), retinal vasculitis and positive anti dsDNA. She was started on systemic steroid and Azathioprine. Symptoms became better and steroid was gradually tapered. However 3 months later she developed relapse of lupus activity with worsening lupus nephritis (active urinary sediments, heavy proteinuria up to 7.6g/day), cytopenia and high anti dsDNA titre. ESR was high but CRP was normal, a pattern commonly seen in active lupus. Azathioprine was switched to Mycophenolate Mofetil without much success. CXR was performed which showed prominent horizontal fissure of right lung. HRCT thorax scan, to our surprise, showed features of early TB reactivation. She had absolutely no respiratory symptom. Girl’s TST and CXR before commencement of steroid were negative. Gamma Interferon Releasing Assay (IGRA) was positive. Bronchoscopy finding was normal. Only the BAL of the RUL bronchus grew Mycobacterium Tuberculosis.

Upon further questioning the parents recalled that the girl's uncle died from pulmonary TB 4 years ago. She stayed with his family during a summer holiday 8 years ago.

After commencing anti TB treatment, her SLE activity remitted with resolution of proteinuria and cytopenia, allowing steroid tapering.

## Results

Table 1

**Table 1:** Cumulative results

Items and reference value	Before anti TB treatment, on Prednisolone, Mycophenolate	After Anti TB treatment, on Prednisolone, Mycophenolate
Hb d/dL	7.7	11.6

Wcc 10^9	2.7	5.3

C3 g/l (0.9-1.8)	0.21	0.98

C4 g/l (0.1-0.4)	<0.02	0.2

Cr unol/l (49-83)	82	44

Albumin g/l (35-52)	23	39

24 hour urine total protein g/day (<0.1)	7.6	0.3

## Conclusion

This case illustrated the need of considering TB infection in endemic region as a cause for persistent SLE activity despite treatment, even when the patient is "asymptomatic" for TB. It is plausible the onset SLE in this patient was actually triggered by the TB reactivation. This case also argues whether in endemic area doing IGRA before steroid commencement would have picked up the latent TB infection as the contact history may not be always reliable.

## Disclosure of interest

None declared
